# A comparative study of Lumbar Decompression and Fusion with Internal Fixation versus Simple Decompression in elderly patients with two-segment Lumbar Spinal Stenosis

**DOI:** 10.12669/pjms.37.1.2287

**Published:** 2021

**Authors:** Pengfa Tu, Shuo Cao, Chenyang Jiang, Chong-chao Yan

**Affiliations:** 1 Pengfa Tu, Department of Orthopaedics, Baoding First Hospital, Baoding, Hebei, 071000, P.R. China; 2 Shuo Cao, Color Doppler Ultrasound Room, Baoding First Central Hospital, Baoding, Hebei, 071000, P.R. China; 3 Chenyang Jiang, Department of Orthopaedics, Baoding First Hospital, Baoding, Hebei, 071000, P.R. China; 4 Chong-chao Yan, Department of Orthopaedics, Baoding First Hospital, Baoding, Hebei, 071000, P.R. China

**Keywords:** Decompression and fusion with internal fixation, Lumbar spinal stenosis, Simple decompression

## Abstract

**Objective:**

To investigate and compare the effect of decompression and fusion with internal fixation vs. simple decompression in the treatment of elderly patients with two-segment lumbar spinal stenosis (LSS) in perioperative and postoperative follow-up periods.

**Methods:**

Twenty-eight elderly patients with two-segment LSS admitted in Baoding First Hospital between Mar. 2017 and Jan. 2018 were retrospectively analyzed. Fifteen patients who underwent simple decompression were included in the simple decompression group, and 13 who underwent decompression and fusion with internal fixation were included in the decompression-fixation group. The general data and perioperative conditions including wound complications, operation time, blood loss, and VAS (legs) and JOA score were analyzed and compared between the two groups.

**Results:**

There was no significant difference in postoperative leg pain (VAS) between the two groups, and a statistically significant difference in JOA score was found between the two groups one month after the operation. The operation time, length of stay, and blood loss in the decompression-fixation group were significantly different from those in the simple decompression group and no significant difference in wound complications was observed between the two groups.

**Conclusion:**

There is no significant difference in leg pain relief in elderly patients with two-segment LSS when treated with decompression and fusion with internal fixation or simple decompression. Simple decompression is associated with less intraoperative injuries, better postoperative functional recovery, and reduced hospital stay.

## INTRODUCTION

Lumbar Spinal Stenosis (LSS) refers to a series of clinical symptoms induced by all-cause osteoporosis or fibrous tissue hyperplasia leading to the sagittal diameter of the spinal canal or nerve-root canal smaller than normal, stimulation or compression of the spinal nerve root or cauda equine. The most common clinical manifestation is neurogenic intermittent claudication.^1^ LSS is the most common indication for lumbar surgery in the elderly population and two main surgical methods are used in the treatment of LSS, i.e., simple decompression and decompression and fusion with internal fixation. In the present study, general information, surgical status, VAS and JOA scores of elderly patients with two-segment LSS in admitted patients to our hospital were compared and analyzed to investigate the effects of the two surgical methods in the treatment of LSS.

## METHODS

In this retrospectively analyzed study, 28 elderly patients with two-segment LSS admitted to Baoding First Hospital between Mar. 2017 and Jan. 2018 were selected. 15 patients received simple decompression surgery were included in the simple decompression group, and 13 received decompression and fusion with internal fixation were included in the decompression-fixation group. General information of the two groups was shown in Table-I.

**Table-I:** General information of the two groups.

Items observed	Decompression group(n=15)	Decompression-fixation group (n=13)	statistics	P-values

Age(years,?x±S)	68.466±4.033	67.307±4.750	*t*=0.698	<0.05
F/M(cases)	9/6	6/7		<0.05^a^
Duration of disease(months)	43.733±11.259	45.461±9.051	t=-0.443	<0.05
Hospital stay (days)	10.733±2.711	15.384±2.567	t=-4.639	<0.01

a Note:Fisher exact probability.

**Table-II:** Surgery parameters of the two groups.

*Items observed*	*Operation time (mins)*	*Blood loss (ml)*	*Wound complications*	

			Yes	No
Decompression group(n=15)	107.800±15.335	305.666±52.140	1	14
Decompression-fixation group(n=13)statistics	159.153±19.582	563.000±96.803	4	9
	t=-7.778	t’=-8.568		
*P-*values	<0.01	<0.01	<0.05 a	

a Note:Fisher exact probability.

### Ethical approval

The study was approved by the Institutional Ethics Committee of Baoding First Hospital at January 6, 2019, and written informed consents were obtained from all participants.

### Inclusion criteria:

1. Symptoms associated with LSS, such as nerve and spinal cord compression symptoms.
2. Image evidence supporting LSS.
3. Ineffective conservative treatment for at least 3 months.
4. Less than or equal to one year of follow-up and complete follow-up data.
5. Age > 60 years.

### Exclusion criteria:

1. Other spinal disorders such as spinal tumors, spinal infections, and scoliosis.
2. Previous lumbar surgery.
3. History of lumbar trauma.
4. Unstable and slipped lumbar spine.

### Surgical Procedures:

### Simple decompression surgery

Patient was placed in the prone position, and then disinfected and draped. Segments for surgery were localized and a longitudinal incision was made. The patient’s skin and subcutaneous tissue were cut by layers to expose the vertebral plate.Vertebral plate fenestration and decompression were performed. The hypertrophic ligamentum flavum was removed, and the lateral recess and nerve root canal were decompressed until the dural sac became loose and the nerve root was no longer compressed. A drainage tube was placed and the incision was closed by layers.

### Decompression and fusion with internal fixation

Patient was placed in the prone position, and then disinfected and draped. Segments for surgery were localized and a longitudinal incision was made. The patient’s skin and subcutaneous tissue were cut by layers to expose the vertebral plate.Pedicle screw was implanted through the pedicle of vertebral arch of the surgical segment. After the internal fixation position was confirmed under roentgenoscopy, two connecting rods were used and properly opened to lock the screw. The spinous process, bilateral vertebral plates and inferior articular process were removed with a bone rongeur. The fibrous ring of the diseased disc was opened, the diseased disc and the endplate was removed. The lateral recess and nerve root canal were decompressed.after the intervertebral space was rinsed with saline, bone graft was implanted. Then the screw was loosened and tightened again after proper compression. A drainage tube was placed and the incision was closed by layers.

### Measurements

General information of patients included age, sex, duration of disease and days of hospital stay. Surgical parameters included the operation time, intraoperative blood loss, and wound complications (including wound fat liquefaction, hematoma, and wound infection). Leg pain was evaluated for all patients before, and one week, 1month, three months, six months and one year after operation at follow-up visits using the visual analogue scale (VAS). Back pain and spinal cord function for all patients before, and one week, one month, three months, six months and one year after operation at follow-up visits using the Japanese Orthopedic Association Score (JOA, 29 points maximum).

Statistical analysis was conducted by SPSS 21.0 software. All data were presented as mean ± standard deviation (tχ ± s) and significance level was set as P= 0.05. Normal distribution was analyzed and measurement data with normal distribution was compared with between groups by independent sample t test was used for measurement data that conformed to normality; and measurement data without normal distribution and homogeneity of variance were analyzed by non-parametric rank sum test. Numeration data were compared by chi-square test. Intra-group comparison of repeated measurement data was analyzed using the repeated measure ANOVA.

## RESULTS

A total of 15 patients, nine males and six females, were included in the simple decompression group, with a mean age of 68.466±4.033 (62-76) years. Duration of disease was 43.733±11.259 months and the length of hospital stay was 10.733±2.711 days. Thirteen patients, six males and seven females, were included in the decompression-fixation group. The mean age of these patients was 67.307±4.750 (61-77 years), duration of disease was 45.461±9.051 months, and the length of hospital stay was 15.384±2.567 days. There were no significant differences in sex, age (t = 0.698), duration of disease (t = -0.443) between the two groups (P > 0.05), and the length of hospital stay was statistically significant between the two groups (t = -4.639, P < 0.01).

The mean operation time was 107.800±15.335 minutes and the blood loss was305.666±52.140ml in the simple decompression group with wound complications in one patient (6.667%). The mean operation time was 159.153±19.582 minutes and the blood loss was 563.000±96.803ml in the decompression-fixation group with wound complications in four patients (30.769%). Statistically significant differences in the operation time (t = -7.778) and blood loss (t ‘=-8.568) were observed between the two group, and no significant difference in wound complications was found between the two groups (P> 0.05). The VAS score showed no statistically significant difference between the two groups at each time point (P> 0.05). The VAS scores in both groups at each time point after the operation were statistically different from the corresponding values before the operation (P<0.05). The JOA score demonstrated that the simple decompression group was better than the decompression-fixation Group-1 month after the operation and the difference was statistically significant (P<0.05). The JOA scores in both groups at each time point after the operation were statistically different from the corresponding values before the operation (P<0.05).

**Table-III:** VAS score of the two groups.

	Pre-op	One week post-op	One month post-op	Three months post-op	Six months post-op	1 year post-op	F values^a^	P values

Decompression group	6.533±1.505	3.466±1.187^a^	2.933±1.099^a^	2.266±1.032^a^	1.733±1.032^a^	1.400±0.985^a^	40.438	<0.01
Decompression-fixation group	6.692±1.377	4.153±1.214^a^	3.000±1.224^a^	1.846±1.214^a^	1.230±0.926^a^	1.230±1.091^a^	55.708	<0.01
*t* values	-0.290	-1.512	0.152	0.991	1.346	0.431		
P values	<0.05	<0.05	<0.05	<0.05	<0.05	<0.05		

Note:

a intragroup ANOVA for repeated measurements.

**Table-IV:** JOA score of the two groups.

	Pre-op	One week post-op	One month post-op	Three months post-op	Six months post-op	One year post-op	F values^a^	P values

Decompression group	14.866±2.526	18.666±2.526^a^	21.600±2.772^a^	22.600±1.549^a^	24.000±1.927^a^	25.000±1.414^a^	68.048	<0.01
Decompression-fixation group	15.307±1.493	19.615±1.363^a^	19.230±1.363^a^	21.153±1.625^a^	20.769±1.832^a^	22.000±2.309^a^	26.427	<0.01
*t* values	t’=-0.553	t=-0.956	t’=2.927	t=2.408	t=4.525	t=-4.209		
P values	<0.05	<0.05	<0.01	<0.05	<0.01	<0.01		

a Note:intragroup ANOVA for repeated measurements.

## DISCUSSION

LSS is a progressive degenerative disease in the structure of intervertebral joints and ligaments inducing spinal or/and neural root canal stenosis. LSS is the most common indication for spinal surgery in the elderly population. Surgical treatment can relieve nerve compression, prevent the disease process, and improve the quality of life. Therefore, surgical treatment is more effective than non-surgical conservative treatment in patients with severe LSS.^2^ With the aging of the population and the advance of technology, more patients are willing to use surgical treatments for LSS.^2^,^3^

Two main surgical methods are currently available for LSS, i.e. the simple decompression surgery and the decompression and fusion with internal fixation^4^, and the use of the latter is increasing at the discretion of surgeons.^5^,^6^ There is much controversy over the application of decompression and fusion with fixation for LSS, especially elderly patients. The prevailing theory supporting the use of decompression and fusion with fixation for LSS without spinal instability is that this surgery can sufficiently improve the symptoms of back pain. Some researchers believe this cannot be achieved via simple decompression. However, studies have shown that the clinical efficacy of simple decompression and decompression and fusion with internal fixation for LSS is not statistically different when lumbar is stable, and the risk of postoperative instability is not increased.^7^-^9^ In our present study, no statistical difference in the clinical effect of the two surgical methods on the symptoms of patients’ lower limbs was found. The long-term follow-up revealed that the functional recovery of patients in the simple decompression group was better. Perioperative complications such as pneumonia, respiratory failure, and cerebral infarction also increased with the increased invasive operation, prolonged operation time, and extensive tissue dissection.^6^,^10^,^11^ It was found in this study that the days of hospital stay were prolonged and the blood loss was increased in the decompression and fusion with fixation group compared with those of the simple decompression group. With the increase of the operation time and the electrosurgical stimulation, the tissue was more seriously damaged, leading to an increased risk of postoperative wound infection. Although no statistically significant difference in wound complications was noted, the proportion of patients with wound complications increased in the combined therapy group. On this basis, the surgical risk of the combined therapy in some elderly patients with severe underlying diseases remains high.^12^,^13^

Studies have reported that the risk of lumbar spondylolisthesis is not increased after simple decompression surgery in LSS patients with no lumbar segmental instability.^7^,^14^ Epstein et al. showed that internal fixation instruments increased the risk of adjacent segment disease in the elderly.^15^ Some surgeons correct all anatomical abnormalities to avoid future symptoms. The theory behind this “preventive” approach, however, has not been proven and the risks of greater complications should be weighed against the longer-term benefits of extensive surgery. Therefore, care should be taken to consider whether simple decompression is sufficient, whether decompression alone is sufficient or it is possible to retain posterior stable structures such as the lumbar facet joint or interspinous ligament. Surgical segments and materials used for implantation need to be carefully evaluated in the case of vertebral instability requiring fusion and internal fixation to reduce soft tissue damage and facilitate postoperative recovery.

### Limitations of the study

It includes the small sample size and the lack of longer-term follow-up data. Studies with large sample sizes and long-term follow-up data are needed.

## CONCLUSION

It was confirmed in this study that there is no significant difference in the therapeutic effect in elderly patients with two-segment LSS when treated with simple decompression or decompression and fusion with internal fixation. Meanwhile, simple decompression is associated with less soft tissue damage (which is conductive to long-term rehabilitation and functional reconstruction), shortened operation time, reduced the blood loss and decreased hospital stay.

### Authors’ Contributions:

**PT and CCY** designed this study and prepared this manuscript.

**SC, CJ and** **FY** collected and analyzed clinical data.

**JXH and** **XBL** significantly revised this manuscript and are responsible for integrity of the study.
